# Adolescent brain maturation and the neuropathological effects of binge drinking: A critical review

**DOI:** 10.3389/fnins.2022.1040049

**Published:** 2023-01-17

**Authors:** Samuel Tetteh-Quarshie, Mary-Louise Risher

**Affiliations:** ^1^Department of Biomedical Science and Research, Joan C. Edwards School of Medicine, Marshall University, Huntington, WV, United States; ^2^Neurobiology Research Laboratory, Hershel ‘Woody’ Williams Veterans Affairs Medical Center, Huntington, WV, United States

**Keywords:** alcohol, binge drinking, adolescence, maturation, neurocognitive

## Abstract

Adolescence is a transitional stage marked by continued brain development. This period is accompanied by physical and neurochemical modifications in the shape and function of the hippocampus, prefrontal cortex, and other limbic system structures. Brain maturation during adolescence, which is typically governed by intrinsic factors, can be dramatically altered by environmental influences such as drugs and alcohol. Unlike many other addictive substances, binge drinking is very common and normative among teenagers and young adults. This repeated pattern of excessive alcohol consumption in adolescents has been shown to cause behavioral changes and neurocognitive impairments that include increased anxiety, risky decision-making, and learning deficits, which could lead to the development of alcohol use disorder (AUD). This manuscript highlights factors that lead to adolescent binge drinking, discusses maturational changes that occur in an adolescent’s brain, and then evaluates the effect of adolescent alcohol consumption on brain structure, function, and neurocognitive abilities in both human studies and animal models. The impact of gender/sex and COVID-19 are briefly discussed. Understanding the factors that promote the onset of adolescent binge drinking and its undesirable consequences could serve as a catalyst for developing therapeutic agents that would decrease or eradicate the damaging effects of alcohol on an adolescent brain.

## 1. Introduction

Adolescence is a developmental period, evidenced by distinct physical, structural, and behavioral changes (Spear, 2016). This transitional period is usually split into early, intermediate, and late stages in both humans and rodents (Salmanzadeh et al., 2020). Even though characterizing the exact start and end of adolescence is challenging, there are distinct developmental and behavioral qualities seen during each stage (Spear, 2000). Adolescence is also a time of increased incidences of psychological disorders such as depression, and anxiety which occur during early to mid-adolescence, and schizophrenia which can emerge during late adolescence to adulthood (Paus et al., 2008). Substances of abuse such as alcohol and drugs are also frequently initiated at this age, with reports showing that cases of alcohol misuse and addiction are high during the middle and latter stages of adolescence and the transition into young adulthood (Anderson et al., 2010).

Alcohol, a widely used recreational drug, is consumed during adolescence and young adulthood by many Americans. It is estimated that excessive alcohol use accounts for close to 95,000 deaths in the United States every year (Centers for Disease Control and Prevention, 2020). This makes alcohol the third leading cause of preventable deaths in the United States, behind tobacco- and obesity-induced deaths (Mokdad et al., 2004). In a national survey conducted by Sacks et al. (2015), it was reported that in 2010 alone, excessive drinking cost the U.S. about $250 billion, or $2.05 per drink. One of the major reasons for the high death toll and economic impact is the alarming rate of alcohol use among American adolescents that has the potential to escalate over time. For example, reports from the 2020 National Survey on Drug Use and Health (NSDUH) showed that about 8.2% of adolescents between the ages of 12 to 17, and about 52% of adolescents between the ages of 18 to 25 indicated that they had used alcohol in the past 30 days (SAMHSA, 2020). This rate of alcohol consumption was anticipated to increase exponentially following the COVID-19 pandemic. However, recent findings demonstrate that the pandemic had mixed results on alcohol consumption. For instance, in a study that surveyed Swiss university students (i.e., mean age = 27.0 years) between April 2020 and June 2021, 20% of the sampled population reported an increased in alcohol consumption, with 26% indicating that they engaged in binge alcohol consumption (Zysset et al., 2022). Conversely, in two separate studies conducted by Bonar et al. (2021) in adolescents with a mean age of 18.05 years, and Bollen et al. (2021) in adolescents with a mean age of 22.10 years, the authors reported reduced alcohol consumption. The discrepancy in these findings could be due to the different age groups analyzed in these studies and increased parental monitoring in the younger cohorts. Further data from longitudinal studies are continuing to be analyzed and will provide valuable information regarding the local and global impact of the COVID-19 pandemic and the potential impact that variations on regulatory lock-down procedures has had on drinking outcomes. For further review on the impact of COVID-19 on adolescent binge drinking see (White et al., 2020; Charles et al., 2021; Rogés et al., 2021; Vasconcelos et al., 2021).

Binge drinking is defined by the National Institute on Alcohol Abuse and Alcoholism (NIAAA), as consuming a significant amount of alcohol such that blood alcohol concentration rises to 0.08 g/dL or at least 0.08% (NIAAA, 2004). In rodent studies, binge-like drinking is modeled by repeated intermittent episodes of alcohol exposure either through intragastric gavage, self-administration, or vapor inhalation exposure, followed by withdrawal (Becker and Lopez, 2004; Hiller-Sturmhöfel and Swartzwelder, 2004; Vargas et al., 2014). This form of alcohol consumption has been linked to acute loss of motor coordination and poor cognitive performance (Fillmore, 2007; Lees et al., 2020). However, it is worth noting that the quantity of drinks that constitute binge drinking differs between men and women. Hence, the definition of binge drinking for women constitutes ingesting four or more standard alcoholic drinks, while for men it constitutes ingesting five or more standard drinks within a 2-hour interval (Chung et al., 2018). Additionally, the frequency and amount of alcohol consumed by adolescents and adults differ per occasion. The quantity of alcohol consumed per occasion is higher in adolescents than in adults, even though, they do so less frequently (Chung et al., 2018). Results from longitudinal studies show that underage drinkers normally drink 4 to 5 standard drinks (i.e., one standard drink contains 14 grams of alcohol) at a sitting. Compared to adults, this number is almost twice what adults consumed on average (Chung et al., 2018). Even though the rate at which alcohol use is initiated is comparable in boys and girls, the rate at which drinking becomes a problem, possibly leading to AUD during adulthood is more prevalent in males than in females (Young et al., 2002; Smith et al., 2021). Binge alcohol consumption during adolescence is a major public health concern that is associated with unintended injuries, suicidal thoughts, severe AUD, and neurocognitive deficits (Crego et al., 2009; Parada et al., 2012; Mota et al., 2013; White and Hingson, 2013; López-Caneda et al., 2014; Carbia et al., 2017; Kanny et al., 2018; Lees et al., 2019). The high prevalence of binge drinking and the increasing evidence of binge alcohol-induced cognitive dysfunction has led studies to examine whether adolescents are more vulnerable to the neurotoxic effects of alcohol because of the structural and functions changes that occur during adolescence (Jones et al., 2018). Therefore, the central thesis of this critical review is to highlight factors that contribute to adolescent binge drinking, discuss maturational changes that occur in an adolescent brain, and evaluate the effect of adolescent alcohol exposure on neurocognitive abilities in humans and animal models with a focus on prefrontal cortex and hippocampus.

To present a balanced review of both human and rodent models on a topic that has received considerable attention, the following criteria influence the inclusion and exclusion of studies in this review. Firstly, studies were included to the introduction if they provided broad overview on binge drinking and its potential impact on adolescent health. Due to the lack of recent studies on social factors that influence adolescent binge drinking, studies included in section “Social factors that influence adolescent binge drinking: peers, parents/guardians, and society” were mainly review articles and observational studies published in the last 30 years. However, given the huge interest in alcohol research and the appreciable work done over the years, pre-clinical and longitudinal/cross-sectional studies were included in sections “Brain maturation during adolescence, Effect of adolescent binge drinking on brain structure and function, Effect of adolescent binge drinking on higher cognitive abilities” if they highlighted brain maturation during adolescence and provided extensive review and/or empirical data on the effect of binge alcohol exposure on neurocognitive abilities in humans and animal models. Finally, studies that had major flaws in their experimental designs, unclear research aim, conference presentations, and unpublished manuscripts were excluded from this review. Again, this decision was influenced by the tremendous progress made in the field of study over the last decade.

## 2. Social factors that influence adolescent binge drinking: peers, parents/guardians, and society

Physiologically, adolescence marks a period of physiological and psychosocial change which raises one’s desire to make their own decisions. In addition to the physiological changes that occur during this stage, adolescents also turn to their peers for support and guidance instead of their parents (Brown et al., 2008). This is because it has been shown that peer-directed interactions are rewarding especially during adolescence (Douglas et al., 2004). These interactions are important for developing new social skills and support networks (Harris, 1995) which may ease the transition to adulthood, especially when an adolescent is away from the family (Spear, 2010). During this transitional period in the life of an adolescent, there is an increased focus on peer-directed social relationships, along with rising conflicts with parents, and elevations in risk-taking and sensation-seeking behaviors (Spear, 2010). In humans, however, the shift from parental dependence to peer approval or independence could also promote daring behaviors, such as engaging in binge alcohol consumption (Steinberg, 2008; Schriber and Guyer, 2016). For instance, data from the National Center for Statistics and Analysis, showed that in 2020 binge drinking contributed to a higher incidence of unintended injuries through alcohol-induced increase in risky behavior and alcohol-impaired driving which resulted in about 12,000 deaths–a 14% increase from 2019 (NCSA, 2020). Therefore, factors influencing risky behaviors in adolescents warrant thorough investigation. Among these, peer pressure has been identified as one of the key factors that compel adolescents to engage in various risky behaviors such as binge drinking.

Peers, examples of social facilitators, can invoke risk-taking behaviors such as alcohol consumption which usually occurs during peer gatherings (Mayer et al., 1998; Sieving et al., 2000; Friese et al., 2013). These social contexts create the platform for adolescents to engage in binge drinking because under such settings adolescents may be under the impression that accepting alcohol offered by peers or drinking more with peers would help them gain social approval and acceptance (Hallett et al., 2014). In addition to peers providing access to alcohol, factors such as parental influence and places where alcohol consumption occurs are also possible contributors to adolescent binge drinking. For example, it is more likely for adolescents to engage in binge drinking at pubs or places where there is no parental supervision. These factors have been proposed as possible contributors to the high incidences of alcohol-related violence in adolescents (Mair et al., 2015).

Parents, on the other hand, have a commanding influence on how adolescents develop or behave, as well as whether they misuse alcohol or not (Lamborn et al., 1991; Kaynak et al., 2014). Parental monitoring and involvement in an adolescent’s life is important because children who feel loved and supported most often are less likely to exhibit risky behaviors such as binge drinking. By studying the influence of parents on adolescent development, it has been determined that adolescents whose behaviors are well-monitored by their parents are less likely to engage in alcohol and other drug use (Dishion and Loeber, 1985; Borawski et al., 2003; Habib et al., 2010). Conversely, it is reported that parents who depend on alcohol tend to offer little or no parental guidance–an act that increases the chances that an adolescent may start using alcohol (Windle, 1996). In scenarios where parents drink excessively and fail to provide the much-needed parental guidance, adolescents may be compelled to associate with friends who engage in activities such as binge drinking. Additionally, parents who are fond of drinking alcohol tend to be lenient and tolerate adolescents who drink more or misuse other substances thus normalizing this behavior (Windle, 1996; Gilligan et al., 2012).

Normalizing and perceiving alcohol as a hallmark of adulthood in today’s society has unintended consequences on adolescents and hence, could serve as a contributing factor to adolescent binge drinking. Even though western societies have experienced massive changes in the laws and regulations surrounding alcohol advertisement, it is hard to argue against the overshadowing influence of alcohol advertisements on different platforms as contributing factors to adolescent alcohol exposure (Bonnie and O’connell, 2004; Anderson et al., 2009). The lack of universal policies and regulations governing alcohol advertisement make it is easy for curious adolescents to fall prey to some of these overshadowing alcohol commercials on a tv set or as portrayed in music videos. These limitations have raised concerns about the alcohol industry in general and the type of information they disseminate to the public, especially to adolescents. For example, in a study that examined the transparency and accuracy of the information propagated by 27 alcohol industries on the association between alcohol and cancer, Petticrew et al. (2018) found a significant misrepresentation of evidence on the risk of developing cancer following alcohol consumption. This is particularly troubling, since most of these industry players are key stakeholders in developing alcohol-related policies in some countries. Given these shortcomings, it is important to implement public health policies that would regulate alcohol advertisement and/or restrict underage teens from entering public events where alcohol is easily accessible, as well as scrutinize the information disseminated by the alcohol industry. Another major obstacle that hinders the effort to mitigate adolescent binge drinking is the level at which alcohol is accepted and regarded as a norm in today’s society. This notion certainly influences underage alcohol consumption. It comes as no surprise that in communities where policies on underage drinking are strictly enforced and youths receive proper parental guidance, incidences of underage drinking are scarce (Bonnie and O’connell, 2004). In addition to these environmental factors, college and university campuses are ideal environments where the use of alcohol tends to be tolerated and encouraged by students. For example, the development of new peer networks in colleges, the low cost of alcohol on or around campuses, and the rate at which campus events involve alcohol are all factors that can increase the tendency of a university student to engage in alcohol consumption (Borsari and Carey, 2001; Weitzman et al., 2003; Hallett et al., 2014; Roberson et al., 2018). Even though others argue that pragmatic steps and strict policies have been enacted by some colleges and universities, these environments remain ideal for alcohol use by adolescents (Bonnie and O’connell, 2004). This is evident in a nationwide survey of college students (i.e., between the ages of 18 to 22 years), where about 53% of the students surveyed indicated that they had consumed alcohol within the past month, and 33% reported to have been involved in binge drinking during that same time frame (SAMHSA, 2019). As stated previously, this level of drinking often leads to an increased prevalence of alcohol-related accidents such as motor and vehicle crashes (Hingson et al., 2017), and behavioral problems such as fighting (Swahn et al., 2004), and unsafe sexual practices (Hingson et al., 2003; Moure-Rodríguez et al., 2016) including unintentional sex with and without protection (Rehm et al., 2012; Chaney et al., 2016). NIAAA estimates that close to 700,000 adolescent students are assaulted by their colleagues who engage in excessive drinking (Hingson et al., 2005). With more recent data showing that for every five college women, one is likely to be sexually assaulted while in college (Muehlenhard et al., 2017). Unfortunately, most of these cases are alcohol or other substance of abuse-related (Lawyer et al., 2010; Carey et al., 2015).

Risk-taking behaviors in adolescents, driven by these contextual factors, have been hypothesized to provide adaptive functions, such as providing opportunities to explore adult behaviors which include alcohol consumption. Taken together, it is evident that the social factors highlighted here increase peer-directed interactions and the seeking of novel and exciting stimuli (Spear, 2010), such as alcohol, which may enhance incidences of binge alcohol consumption (Weitzman et al., 2003) and alcohol-induced injuries in adolescents (Swahn et al., 2004). Here, we briefly explore maturational changes that take place in human and rodent adolescents and how these processes are impacted by binge drinking.

## 3. Brain maturation during adolescence

As stated previously, adolescence is a transitional period that results in many neurobiological and physiological changes in the brain (Spear, 2016). During this period, there are also neurodevelopmental changes in synaptic plasticity and neural connectivity, that are ongoing and important for brain refinement and specialization (Giedd, 2004; Carbia et al., 2018; Lees et al., 2019; Squeglia, 2020). These processes are critical because maturing connections among brain regions enhance brain network integration and complexity (Pascual et al., 2018). Tremendous progress has been made in the field of neuroscience and addiction to understand how the brain functions and how insults such as alcohol affect specific regions of the brain, especially during adolescence (Arain et al., 2013; Chwedorowicz et al., 2017; El Marroun et al., 2021; Walker et al., 2021). Two of the major maturational changes that happen during adolescence that have received considerable attention are alterations in both white and gray matter. Physiologically, it has been determined that gray matter volume decreases during adolescence–a maturational phenomenon that enhances synaptic pruning and myelination in the cortex (Giedd, 2004; Gogtay and Thompson, 2010; Pfefferbaum et al., 2016; Dow-Edwards et al., 2019; Sakai, 2020; El Marroun et al., 2021). It has also been shown that during adolescence, there is a continuous growth of white matter fibers–a process that enhances communication between different brain regions (Giedd, 2004; Lebel and Beaulieu, 2011; Yap et al., 2013; Dow-Edwards et al., 2019). These changes have been reported in different brain regions. For example, the prefrontal cortex, an area that mediates critical, complex cognitive abilities has been shown to have decreasing gray matter volume during the transition from adolescence to adulthood (Giedd, 2004). Similarly, changes in subcortical brain structures have also been identified. For instance, through magnetic resonance imaging study it has been shown that the size of subcortical regions such as the putamen and caudate decreases throughout adolescence. Inversely, subcortical regions such as the hippocampus and amygdala are shown to first increase in size during puberty, with slowing but continual growth observed into adolescence (Østby et al., 2009).

In addition to structural remodeling, reports indicate that neurochemical maturation also occurs during these formative years. For example, it has been determined that during adolescence, there is reorganization in the dopaminergic system involved in reward and incentive processing (Wahlstrom et al., 2010). Specifically, during adolescence, it has been demonstrated that dopamine fibers continue to increase in density in the medial prefrontal cortex (Naneix et al., 2012; Willing et al., 2017). The continual increase of these fibers potentially makes adolescents vulnerable to positive and negative influences (Hoops and Flores, 2017). This finding supports a previous report by Andersen et al. (2000) that shows that dopamine receptor expression increases maximally during adolescence in cortical and subcortical regions. Another brain region that has been heavily explored because of its involvement with reward and sensation-seeking is the nucleus accumbens (NAcc). Neuroimaging studies have also shown that NAcc is very sensitive during the formative years. It has been discovered that changes in reward sensitivity during adolescence are partly due to decreasing dopamine signaling; possibly explaining why adolescents may engage in sensation-seeking and risky behaviors (Arain et al., 2013). Even though the exact molecular mechanisms driving sensation-seeking and the onset of early alcohol use are not clearly understood, a recent study by Morales et al. (2019) shows that NAcc possibly mediates individual differences in sensation-seeking during adolescence.

As stated previously, during adolescence there are ongoing and significant changes to the projecting neuronal circuitry between brain regions geared toward improving cognition. For example, it has been shown that the continuous growth and development of these circuits enhances cognitive abilities such as multitasking, problem-solving, and the ability to process complex information (Arain et al., 2013). Developing these higher cognitive abilities as one transitions from childhood to adolescence requires a healthy and functioning brain, but since an adolescent brain is still undergoing maturation, they are more vulnerable to many insults such as alcohol. Based on this assertion, it is obvious that even subtle alterations in structure, thickness of the cerebral cortex, and demyelination due to binge drinking in adolescents could lead to psychological and social consequences (Nagy et al., 2004; Casey et al., 2008). Therefore, to understand how adolescent binge alcohol consumption negatively affects cognition, there is a need for a holistic review of the morphology and function of the hippocampus, prefrontal cortex, and cerebellum. The ensuing section provides a brief overview of human and rodent experiments that examine the effects of binge drinking on brain structure and function during adolescence.

## 4. Effect of adolescent binge drinking on brain structure and function

**FIGURE 1:**
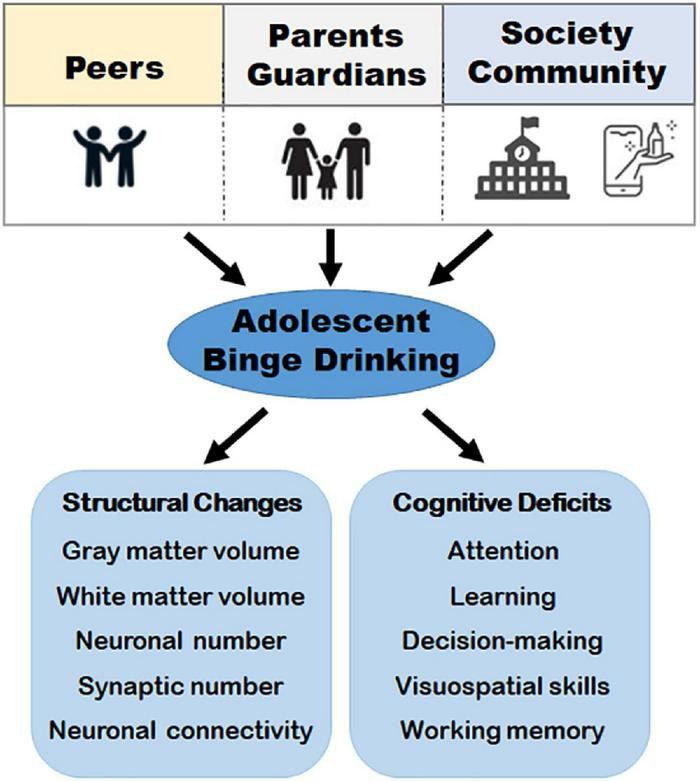
A schematic representation of major factors that contribute to binge drinking and the downstream consequences. Social factors such as peers and social acceptance, family and parental guidance, and the community, combined with internal factors, such as, age, sex hormones, and psychological status can influence binge drinking during adolescence. Binge drinking (i.e., consuming 4 ≥ standard drinks for women and 5 ≥ standard drinks for men in a brief period of time) usually within 2 h, contributes to structural changes such as decreased gray matter volume and white matter density, changes in synaptic number, neuronal loss, and disruption of brain connectivity. These structural changes have been implicated in cognitive deficits such as altered working memory, attention, visuospatial skills, decision-making, and learning deficits in adolescent binge drinkers and young adults.

Brain maturation during adolescence is influenced by inherent factors such as heredity, prenatal and post-natal insults, and extrinsic and environmental factors such as substance abuse (Bolton et al., 2012; Arain et al., 2013; Miguel et al., 2019; Tooley et al., 2021). Among the extrinsic factors, alcohol is highlighted in this review. This is because alcohol binge drinking poses a great challenge to adolescent brain maturation due to how normative it has become among most youths in the United States. This has led to extensive studies on maturational changes and how alcohol alters their development during the formative years (Figure 1).

Changes in brain structure and function following binge drinking in adolescents have been well-examined in human studies using electrophysiology and neuroimaging principles (Table 1). For example, to determine the effect of binge drinking on brain volume and microstructure, a magnetic resonance imaging (MRI) technique was used and demonstrated that adolescents who use alcohol and those with marginal alcohol exposure have different gray matter microstructure volumes (Jacobus and Tapert, 2013). The reduction of gray matter volume observed by Jacobus, aligns with current longitudinal studies that shows that binge alcohol consumption reduces cortical and subcortical gray matter volumes in adolescents (Infante et al., 2022). Cross-sectional human studies reveal that adolescents who engage in binge drinking have different volumes of gray matter in distinct brain areas when compared to non-binge drinking adolescents (Howell et al., 2013; Doallo et al., 2014; Pfefferbaum et al., 2016; Sousa et al., 2019). For example, in their study of adolescent binge drinkers, Pfefferbaum et al. (2016) observed a decrease in the volume of both frontal and temporal lobes in subjects who drank minimal to high volumes of alcohol compared to those who do not consume alcohol. Through the compilation of cross-sectional and longitudinal studies of adolescent binge drinkers (Cservenka and Brumback, 2017; Lees et al., 2020; Almeida-Antunes et al., 2021) it has been demonstrated that adolescents who engage in binge drinking show abnormalities in gray matter volume and deficits in neural activity in distinct brain regions during working memory, verbal learning, and tasks that involve inhibitory control (Cservenka and Brumback, 2017). This evidence stems from magnetic resonance imaging and event-related potentials data from both cross-sectional and longitudinal studies that focus on structural and functional changes in frontal brain regions following binge alcohol consumption. Through these experimental designs, the effects of binge drinking on frontal areas such as the insular cortex, the inferior frontal gyrus (IFG), and the anterior cingulate cortex (ACC) in adolescents during cognitive processes have been investigated (López-Caneda et al., 2012; Xiao et al., 2013; Mashhoon et al., 2014; Suárez-Suárez et al., 2020; Pérez-García et al., 2022). For example, by comparing cortical thickness between binge drinking females and their light drinking counterparts, Mashhoon et al. (2014) found a significant reduction in cortical thickness in the mid-ACC of binge drinkers. Further analysis from their study demonstrated a positive correlation between ACC thinness and alcohol use patterns in the binge drinking group. These findings are further supported by empirical data obtained from event-related potentials studies (López-Caneda et al., 2012). For example, by measuring event-related potentials, López-Caneda et al. (2012) aimed to determine the effect of alcohol on frontal brain areas in young binge drinkers. As part of a longitudinal study that is focused on the prefrontal cortex, López-Caneda et al. (2012) recorded an abnormal brain activity (i.e., hyperactivation of the right inferior cortex) in binge drinkers compared to the control group. This finding aligns with previous experiments conducted by Crego et al. (2010) in which binge drinking university students showed altered prefrontal cortex function during an identical pairs performance task. Specifically, in their experiment, anterior prefrontal cortex activity was significantly reduced in binge drinkers when compared to the control group (Crego et al., 2010). In another study where Kvamme et al. (2016) examined T1-weighted magnetic resonance imaging (MRI) images of college-age binge drinkers and matched healthy volunteers using voxel-based morphometry and covarying the anatomical scans with AUD identification test (AUDIT) scores, Kvamme and colleagues found volumetric changes in the frontal lobe of college-age drinkers. Furthermore, in a recent analysis of data from the National Consortium on Alcohol and NeuroDevelopment, it was demonstrated that cortical gray matter volume was significantly decreased in adolescent binge-drinkers compared to non-binge drinkers (Infante et al., 2022). A complementary study by Pérez-García et al. (2022) examined differences in gray matter morphology between young university stable binge drinkers and a stable control group *via* a cross-sectionally and longitudinally analysis. From their cross-sectional studies, which compared baseline parameters to follow-up, there were no structural abnormalities observed between the two groups at baseline. However, during the follow-up period, Pérez-García and colleagues observed a larger surface area in the left insular in the stable binge drinkers when compared to the stable control group (Pérez-García et al., 2022). While there were no sex-specific differences recorded for the insular cortex, region of interest analysis on structural thickness showed a sex-specific effect where binge drinking males appeared to have smaller right rostral middle frontal gyrus thickness than either the control males or binge drinking females. Similarly, it was evident in their longitudinal study that continuous binge drinking significantly decreased the volume of NAcc. These findings are important because the presence of gray matter enhances learning, motor control, and attention, among others. These findings align with previous data from De Bellis et al. (2005) which aimed to determine how alcohol affects adolescents who have early-onset AUD. Through MRI studies, they reported a decrease in prefrontal cortex volume and smaller volumes of prefrontal cortex white matter in adolescents who had AUD (De Bellis et al., 2005). In a parallel human study, McQueeny et al. (2009) assessed the integrity of white matter in binge-drinking adolescents who had no record of AUD. Similar to the aforementioned study, McQueeny and colleagues found a widespread reduction in fractional anisotropy in major white matter regions–indicating compromise of the integrity of white matter in the sampled population (McQueeny et al., 2009). Even though longitudinal and cross-sectional studies exploring binge drinking-induced hippocampal impairments are not extensively covered in this review, the effect of alcohol on the structure and function of the hippocampus has been identified in adolescents who engage in heavy alcohol consumption. For example, by analyzing MRI brain scans of college freshmen, Meda et al. (2018) found a positive correlation between heavy alcohol consumption and increased hippocampal gray matter volume decline. The finding from this study aligns with previous report showing smaller left hippocampal gray and white matter volumes in adolescents who have AUD compared to their non-alcohol using counterparts (Nagel et al., 2005). Although these two experiments used non-binge drinking patterns, the empirical data support the notion that a variety of alcohol intake models can result in similar outcomes.

**TABLE 1:** Summary of studies exploring structural and functional anomalies in adolescent binge drinkers.

Model	Parameters	Effects	References
Adolescents (*N* = 28; mean age = 16–19 years)	Diffusion tensor imaging analysis of Fractional Anisotropy (FA)	Binge drinking resulted in a widespread reduction of FA in major white matter pathways	McQueeny et al., 2009
Adolescents (*N* = 59; men age = 16–19 years)	MRI analysis of brain morphometry	Binge drinking females showed thicker cortices in the frontal pole, pars orbitalis, medial orbital frontal, and rostral anterior cingulate	Squeglia et al., 2012
Adolescents (*N* = 38; mean age = 22–24)	MRI analysis of voxel-based morphometry	Binge drinkers showed larger ventral striatal gray matter volume	Howell et al., 2013
Adolescents (*N* = 54; mean age = 18–22 years)	High-resolution MRI analysis	Binge drinkers showed a significant reduction in cortical thickness in the mid-ACC	Mashhoon et al., 2014
Adolescents (*N* = 32; mean age = 21–23 years)	Structural MRI analysis of voxel-based morphometry	Binge drinkers showed larger gray matter volume in the left mid-dorsolateral PFC	Doallo et al., 2014
Adolescents (*N* = 674; mean age = 12–21.9 years)	T1-weighted MRI analysis	Binge drinking decreased the volume of both frontal and temporal lobes of the cortex	Pfefferbaum et al., 2016
Adolescents (*N* = 36; mean age = 18–23 years)	T1-weighted MRI analysis	Binge drinkers showed increase nucleus accumbens volume	Sousa et al., 2019
Adolescents (*N* = 166; mean age = 12–21 years)	High-resolution structural MRI analysis	Binge drinkers showed decrease cortical gray matter volume	Infante et al., 2022
Adolescents (*N* = 44; mean age: baseline = 18–19 years; follow-up 20–21 years)	Magnetic resonance imaging analysis	Binge drinkers showed larger insular surface area, and a sex-specific decrease in the right rostral middle frontal gyrus thickness, and in NAcc volume	Pérez-García et al., 2022

It is important to state that it cannot be explicitly determined whether these brain volume reductions in the subjects are due to binge drinking or if the brain volume reduction is a driving factor for high drinking in adolescents and development of AUD. However, the use of rodent models can assist with this obstacle. It has been well-established that effective information processing requires well-structured and functional myelinated nerve fibers (Simons and Trotter, 2007). A study by Montesinos et al. (2015) demonstrated that binge-like alcohol consumption in adolescent mice causes white matter disruption, specifically, ultrastructural myelin sheath disarrangement in the prefrontal cortex and down-regulation of proteins involved in myelination. This finding aligns with previous experiments conducted by Vargas et al. (2014), where reduced myelin density was found in the medial prefrontal cortex of alcohol-exposed adolescent rats (Vargas et al., 2014). These data demonstrate that binge drinking disrupts myelin sheath development. More importantly, since the formation and development of myelin sheaths is important for motor activity and learning (McKenzie et al., 2014; Wang et al., 2020), its disruption due to adolescent binge drinking could contribute to subsequent behavioral deficits in adulthood. In addition to the microstructural changes in gray and white matter volumes induced by binge drinking in adolescents, rodent model experiments have also found structural alterations in the cortex (Vetreno et al., 2017), hippocampus (Risher et al., 2015c), and cerebellum (Vetreno et al., 2016) in adolescent rodents that are exposed to binge ethanol. Vetreno et al. (2017) exposed female adolescent Wistar rats to the adolescent intermittent ethanol (AIE) paradigm from P25 to P55 and found that P80 rats who underwent the drinking paradigm had reduced cerebellar and hippocampal volumes when compared to the controls (Vetreno et al., 2017). These results support previous work conducted with rats that underwent similar adolescent alcohol paradigms and showed neuronal loss within the hippocampus (Crews et al., 2006; Hansson et al., 2010; Risher et al., 2015a,c), and reduced glia number in the medial prefrontal cortex (Koss et al., 2012). Evidence from Broadwater et al. (2014) and Hiller-Sturmhofel and Spear (2018), have demonstrated that alcohol exposure in adolescent rats not only enhances cell death in select brain areas but also decreases neurogenesis, while Morris et al. (2010) demonstrated that adolescent alcohol exposure disrupts the growth of neural stem cells in the dentate gyrus of male Sprague-Dawley rats. Additional rodent studies focusing on the hippocampus support the finding that binge alcohol consumption during adolescence impairs neurogenesis in the dentate gyrus (Lacaille et al., 2015; Vetreno and Crews, 2015; Nwachukwu et al., 2022). This is an important finding because neurogenesis is necessary for developmental processes in select brain regions throughout life, and is important for functions such as learning, memory (Costa et al., 2015), and cognitive flexibility (Toda et al., 2019). Thus, its disruption by alcohol exposure may be a contributing factor to the cognitive decline observed in binge drinkers.

It is possible that disruption of neurogenesis and cell death occurs due to changes in neuroimmune gene expression, potentially leading to alcohol-induced brain damage (Crews et al., 2000; Pascual et al., 2007, 2017; Sanchez-Alavez et al., 2019; Barney et al., 2022). For example, to determine the effects of adolescent ethanol exposure on the regulation of inflammatory markers, Pascual et al. (2007) measured the levels of COX-2 and iNOS (examples of cytokines and inflammatory mediators) in the brain of adolescent rats. They demonstrated that expression of cytokine and inflammatory mediators were significantly increased in the neocortex, hippocampus, and cerebellum of ethanol-exposed rats. In their experiment, the elevations of these mediators correlated with neural cell death and induced neurobehavioral deficits in the rats (Pascual et al., 2007). Interestingly, recent work by Nwachukwu et al. (2022) revealed a sex-specific effect of adolescent ethanol exposure on hippocampal neurogenesis and cytokine release. In their experiment, pro-inflammatory markers such as IL-1β and TNFα were only increased in alcohol-exposed male rats suggesting a sex-dependent differential immune-responsivity to alcohol in adolescence. There are a growing number of pre-clinical studies emerging that support a role for alcohol-induced astrocyte and microglia activation, imbalance of reactive oxygen species, and pro-inflammatory signaling (Crews and Nixon, 2009; Alfonso-Loeches et al., 2012; Risher et al., 2015b; Vetreno and Crews, 2015; McClintick et al., 2018; Melbourne et al., 2021; Peng and Nixon, 2021). Based on these findings, it would be interesting to determine if targeting these non-neuronal cells and related pro-inflammatory signaling pathways is an effective approach to preventing alcohol-induced neuronal cell death. Given that hippocampal integrity is critical for cognitive functions such as learning and memory formation, results from these studies show that alcohol-induced alterations to neurogenesis, cell death, and neuroinflammation could have unintended consequences on brain function (Table 2). For further reading see (Vilpoux et al., 2022).

**TABLE 2:** Summary of studies exploring the effect of adolescent binge-like ethanol intake in rodent models.

Model	Parameters	Effects	References
Male Sprague-Dawley rats (beginning PND35)	Intragastric gavage of single dose ethanol (1.0, 2.5, or 5.0 g/kg, 25% v/v in saline)	Acute ethanol exposure inhibited neural progenitor cell proliferation in the dentate gyrus, forebrain regions, and subventricular zones	Crews et al., 2006
Wister rats (Beginning PND25)	Intraperitoneal administration of ethanol (3.0 g/kg; 25% w/vol), 2-days on/2-days off	Binge-like ethanol administration increased inflammatory cytokine expression, enhanced cell death in the neocortex, hippocampus, and cerebellum	Pascual et al., 2007
Sprague-Dawley rats (Beginning PND28)	Intragastric gavage of ethanol (1.5, 3.0, or 5.0 g/kg; 25% w/vol), 4-day binge model	Repeated binge-like ethanol administration during adolescence enhanced ethanol consumption during adulthood	Maldonado-Devincci et al., 2010
Long-Evans rats (Beginning PND35)	Intraperitoneal administration of ethanol (3.0 g/kg; 25% w/vol), 2-days on/2-days off	Binge-like ethanol administration caused sex-specific decrease in the number of glia cells in the mPFC	Koss et al., 2012
Male Sprague-Dawley rats (beginning PND30)	Intraperitoneal administration of ethanol (3.0 g/kg; 25% w/vol) for 2 consecutive days at 48 h-intervals; followed by operant drinking task	Bine-like ethanol administration during adolescence enhanced motivation for ethanol self-administration during adulthood	Alaux-Cantin et al., 2013
Male Wister rats (Beginning PND 28)	Operant self-administration of sweetened alcohol (8–10% w/v ethanol)	Binge-like ethanol consumption reduced myelin density in the mPFC of adolescent rats	Vargas et al., 2014
Female C57BL/6 Mice (Beginning PND30)	Intraperitoneal administration of ethanol (3.0 g/kg; 25% w/vol), 2-days on/2-days off	Binge-like ethanol exposure increased cytokines and pro-inflammatory mediators levels, resulting in ultrastructural myelin sheath disarrangement in the PFC	Montesinos et al., 2015
Male Sprague-Dawley rats (Beginning PND30)	Intragastric gavage of ethanol (5.0 g/kg, 35% w/v), 2-days on/1-day off, 2-days on/2-days off	Binge-like ethanol exposure decreased the number of mature dendritic spines, and reduced post-synaptic proteins in the hippocampus	Risher et al., 2015c
Male C57BL/6 mice (Beginning PND30)	Intraperitoneal administration of single (2.5 g/kg, twice plus 2 g/kg 2-h apart) or multiple dose ethanol	Multiple binge-like ethanol exposure decreased the number of progenitor cells in the hippocampus with deficits in short-term memory	Lacaille et al., 2015
Male Sprague-Dawley rats (beginning PND35)	2 Intraperitoneal administrations of ethanol (3 g/kg; 20% vol/vol) 9 h apart	Ethanol-treated rats failed to recognize a novel object, an indication of alcohol-induced cognitive deficit	De Ferron et al., 2015
Female Wister rats (Beginning PND25)	Intragastric administration of ethanol (5.0 g/kg; 20% w/vol), 2-days on/2-days off	Binge-like ethanol consumption reduced axial diffusivity (PND80-220) in the cerebellum, hippocampus, and neocortex	Vetreno et al., 2016
Male Wister rats (Beginning PND26)	Two-bottle choice paradigm of ethanol (20%) and water; followed by an operant drinking task (fixed ratio)	Rats exposed to ethanol during adolescence recorded fewer fixed ratio failed response, and consumed more alcohol during adulthood	Amodeo et al., 2017
Wister rats (Beginning PND25)	Intragastric administration of ethanol (5.0 g/kg; 20% w/vol), 2-days on/2-days off	Binge-like ethanol exposed rats showed bilateral thinning of the PFC, with reduced hippocampal and cerebellar volumes	Vetreno et al., 2017
Male Sprague-Dawley rats (Beginning PND25)	Intraperitoneal administration of ethanol (3.0 g/kg; 25% w/vol), 2-days on/-day off	Binge-like ethanol administered rats showed impaired synaptic plasticity with alterations in learning and memory tests	Tapia-Rojas et al., 2018
Male Sprague-Dawley rats (beginning P30)	Intragastric gavage of ethanol (5.0 g/kg, 35% w/v), following AAV microinjection of green fluorescent protein tag	Binge-like ethanol administration induced PFC subregion-specific changes in dendritic spine maturity with changes in astrocyte-neuronal interactions	Walker et al., 2022

Binge-like alcohol exposure results in deficits in learning and memory in rodent models (Tapia-Rojas et al., 2018). Therefore, it should be of no surprise that in addition to neuronal loss, synaptic function in many of these adolescent binge alcohol models, is also impaired. As previously stated, during adolescence, there are constant modifications of synapses and neuronal activities geared toward optimizing higher cognitive abilities (Glantz et al., 2007; Dow-Edwards et al., 2019; Sakai, 2020). Binge-like alcohol consumption during adolescence has been shown to cause aberrant synaptic transmission (Mulholland et al., 2018; Amodeo et al., 2021), in part due to changes in synaptic protein expression and localization of glutamate receptor subunit 2B (GluN2B) in rodent models (Swartzwelder et al., 2016; Wills et al., 2017). For example, by exposing male Sprague-Dawley rats (beginning P30) *via* intragastric gavage to 5 g/kg ethanol during adolescence, Risher et al. (2015c) found that alcohol-exposed rats displayed enhanced synaptic efficacy in the CA1 region of the hippocampus. In their study, the change in long-term potentiation (LTP) was associated with an increase in immature spines that are known to have increased plasticity when compared to more mature dendritic spines. More recent work has demonstrated similar shifts in dendritic spine phenotype within select sub-regions of the prefrontal cortex that are suggested to be associated with a loss of astrocyte support (Walker et al., 2022). Physiologically, the mechanisms modulating alcohol-induced synaptic alterations warrant further study. However, given the findings shown here, it is evident that adolescent intermittent alcohol exposure alters synaptic protein expression and synaptic function, likely contributing to changes in synaptic excitation and subsequent cognitive changes.

Quantification of the measures discussed in the manuscript is rather straightforward in rodent models, however, this becomes more challenging in patient populations. This could be in part due to the unpredictability of the reliance on memory recall and estimation of drink number following intoxication that some retrospective studies employ. Unlike rodent studies where binge drinking, environment, and environmental stressors can be carefully controlled, in longitudinal and cross-sectional studies, it is difficult to truly characterize the true extent of binge drinking in adolescents along with the variety of drivers of such behaviors, thus potentially increasing data variability. Additionally, since most of these retrospective studies rely on answers provided by binge drinkers in surveys, there could be variations in the data collected, especially when adolescents are asked whether they indulge in polysubstance use. These limitations could contribute to the lack of clear parallels between rodent and human studies concerning hippocampal impairment following binge alcohol consumption. Nonetheless, it is worth noting that these retrospective studies have contributed significantly to the field of study and are incredibly valuable to the field of adolescent alcohol in understanding how additional variables contribute to the onset and emergence of AUD. Altogether, the experimental evidence highlighted here suggests that binge drinking during adolescence could modify select brain regions, alter microstructure volume, decrease neurogenesis, affect synaptic integrity, and enhance cell damage and/or cell death, resulting in neuropathological consequences during adulthood. In addition to the structural modifications and consequences highlighted above, the ensuing section reviews how binge alcohol-induced structural modifications can impact higher cognitive abilities in adolescents.

## 5. Effect of adolescent binge drinking on higher cognitive abilities

Research topics focusing on insult to the adolescent brain are of great interest because of the critical transformations that occur during this time-period and clear evidence of adolescent susceptibility to the effects of alcohol. Higher cognitive abilities or executive functions are sets of behaviors that emerge during the early ages of childhood and continue to develop during adolescence and the early twenties (Crone, 2009; Gil-Hernandez et al., 2017), and are important for appropriate integration and adaptation to society (Jurado and Rosselli, 2007). The development of working memory, learning, attention, decision-making, effective planning, cognitive flexibility, inhibition, and self-control are important during maturation. However, binge alcohol consumption during adolescent brain development has been shown to affect these cognitive functions in both rodent and human studies (Goudriaan et al., 2007; Schweinsburg et al., 2010; De Ferron et al., 2015; Carbia et al., 2017; Patte et al., 2017; Tong et al., 2021).

To fully understand how binge alcohol consumption affects these core functions in adolescents (Table 3), studies are currently using encoding cues and different learning tasks to assess human behavior following binge alcohol exposure. In a preliminary human study comparing the performance of verbal encoding tasks in adolescent binge drinkers vs. non-drinkers, Schweinsburg et al. (2010) report that those with a history of binge drinking performed poorly on the task. This finding supports the notion that alcohol can impair the learning or processing of new word pairs. Working memory is another important feature of executive functioning and information processing system that maintains information over a brief period (Tapert et al., 2004). However, adolescents and young adults who engage in binge drinking have been shown to have altered neuronal activity during working memory (Crego et al., 2009, 2010; Squeglia et al., 2011; Campanella et al., 2013; López-Caneda et al., 2013a). For example, in their study involving human subjects, Tapert et al. (2004) identified a negative relationship between brain activity for a task involving visual working memory and alcohol response. In their experiment, healthy adolescents with different drinking habits were recruited and the consequences of acute alcohol were measured through neuropsychological testing. The data collected from this study revealed that adolescents who needed to drink an increased volume of alcohol to achieve intoxication had enhanced stimulation in select brain regions (Tapert et al., 2004). Similarly, by measuring event-related potentials in college freshmen, Crego et al. (2009) found functional differences in a visual task involving a high working memory load. In their experiment, college freshmen who engage in binge drinking required higher attentional effort to carry out the assigned visual task compared to the control group. In another experiment, increased stimulation in the superior frontal gyrus (SFG), inferior parietal lobule (IPL), and supramarginal gyrus was recorded in adolescents who engaged in binge drinking compared to their non-drinking peers under baseline conditions (Squeglia et al., 2012). Given the role of the prefrontal cortex in executive functions, it is important that studies highlight the impact of binge drinking on functions subserved by the prefrontal cortex. For example, by examining the effect of binge drinking on the dorsolateral prefrontal cortex, Parada et al. (2012) report that binge drinking impacts executive control of working memory in college students. A more recent study by Carbia et al. (2017) investigating the effect of binge drinking on verbal episodic memory, demonstrated that adolescents who binge drink showed episodic memory deficits compared to non-binge drinkers. These studies further highlight the vulnerability of the brain to the neurotoxic effects of alcohol.

**TABLE 3:** Summary of behavioral studies exploring the effect of binge drinking in adolescents during cognitive tasks.

Model	Task	Effects	References
**Memory**			
Adolescents (*N* = 27; mean age = 18–23 years)	Neuropsychological assessment of sustained attention and long-term memory (recall tasks)	Binge drinkers performed poorly on the sustained attention and recall tasks	Hartley et al., 2004
Adolescents (*N* = 95; mean age = 18–20 years)	ERP analysis of visual task	Binge drinking affects the execution of a visual task with a high working memory load	Crego et al., 2009
Adolescents (*N* = 24; men age = 16–18 years)	fMRI analysis of verbal encoding task	Binge drinkers showed no hippocampal activation during novel encoding and performed poorly during word pair recall	Schweinsburg et al., 2010
Adolescents (*N* = 95; mean age = 18–20 years)	ERP analysis of identical pair continuous performance task	Binge drinkers showed hypoactivation of the right anterior PFC for matching stimuli during the performance task	Crego et al., 2010
Adolescents (*N* = 95; mean age = 16–19 years)	fMRI analysis of spatial working memory task	Binge drinking females showed less frontal, temporal, and cerebellar brain activation which correlated with poor working memory performances	Squeglia et al., 2011
Adolescents (*N* = 122; mean age = 18–20 years)	Neuropsychological assessment of spatial and verbal working memory	Binge drinkers recorded lower scores during the verbal working memory test	Parada et al., 2012
Adolescents (*N* = 89; mean age: baseline = 18–19 years; follow-up 20–21 years)	Neuropsychological assessment of episodic memory and executive functions	Persistent binge drinkers performed poorly on episodic memory task	Mota et al., 2013
Adolescents (*N* = 32; mean age = 19–24 years)	fMRI analysis of working memory task	Binge drinkers showed higher working memory-related brain activation in the dorsomedial PFC	Campanella et al., 2013
Adolescents (*N* = 155; mean age baseline: 18–19 years; follow-up 24–25 years)	Neuropsychological assessment of verbal episodic memory task	Stable binge drinkers during the follow-up demonstrated verbal episodic memory deficits	Carbia et al., 2017
**Inhibition**			
Adolescents (*N* = 48; mean age: baseline = 18–19 years; follow-up 20–21 years)	ERP analysis of go/no-go task	Binge drinkers showed hyperactivation of the right inferior cortex during response execution and inhibition response	López-Caneda et al., 2012
Adolescents (*N* = 23; mean age = 18–20 years)	fMRI analysis of go/no-go task	Recent binge drinkers showed decreased activation of both dorsolateral and dorsomedial PFC, and ACC during negative inhibitory trials	Cohen-Gilbert et al., 2017
Adolescents (*N* = 67; mean age = 18–19 years)	fMRI analysis of alcohol-cued go/no-go task	Binge drinkers showed increase neural activity in the bilateral inferior frontal gyrus and insula during response inhibition	Suárez-Suárez et al., 2020
**Reward and decision-making**			
Adolescents (*N* = 200; mean age = 16–18 years)	Neuropsychological assessment of Iowa Gambling task	Stable high binge drinkers made less advantageous choices on the IG task which is associated with poor decision-making	Goudriaan et al., 2007
Adolescents (*N* = 28; mean age = 16–18 years)	fMRI analysis of Iowa Gambling task	Binge drinkers perform worse on the IG task and showed higher neural activity in the left amygdala and insula	Xiao et al., 2013
Adolescents (*N* = 48; mean age: baseline = 14–16 years; revisit 16–18 years)	fMRI analysis of Wheel of Fortune (reward processing) task	Binge drinkers showed reduced cerebellar brain activity at revisit during the reward processing task	Cservenka et al., 2015
Emerging adults (*N* = 50; mean age = 21–29 years)	fMRI analysis of a reward-guessing game (the Doors’ task)	Binge drinkers showed enhanced activation in the right and left NAcc during reward processing relative to loss	Crane et al., 2017
Adolescents (*N* = 50; mean age = 21–22 years)	ERP analysis of Iowa Gambling task	Binge drinkers recorded lower total net score which correlates with poor decision-making	Na et al., 2019

In addition to working memory, the association between binge drinking and poor performance in attention, learning, and visuospatial abilities has been examined (Hartley et al., 2004; Squeglia et al., 2011; Mota et al., 2013). For example, Squeglia et al. (2011) found binge-drinking females performed poorly in visuospatial, inhibition, and attention tasks when compared to their non-drinking females. These sex-specific cognitive deficits were attributed to alterations in the cortical thickness of gray matter. The relationship between adolescent alcohol consumption and inhibitory control has also been extensively studied in the last decade. The central hypothesis of these studies is that the inability to inhibit a response (i.e., alcohol use) during adolescence, could promote excessive alcohol consumption (López-Caneda et al., 2013b). To test this hypothesis, Norman et al. (2011) used a go/no-go task to examine response inhibition in relation to alcohol and other substance use in middle schoolers. In their experiment, decreased neural activity in brain regions such as the cingulate gyrus, and left dorsal and medial frontal areas during response inhibition predicted later alcohol use. It has also been determined that college students who engaged in a higher incidence of binge drinking in the last 3 months have altered neural activity in distinct frontal brain regions that are involved in inhibition response (Cohen-Gilbert et al., 2017). Specifically, in this experiment, reduced activation of brain regions such as the ACC, dorsolateral prefrontal cortex, and dorsomedial prefrontal cortex was evident in students with higher recent incidence of binge drinking during the go/no-go inhibitory control task (Cohen-Gilbert et al., 2017). In a more recent study that explored the impact of binge alcohol consumption on brain regions implicated in inhibition response, Suárez-Suárez et al. (2020) recorded enhanced neural activity in brain regions such as the inferior frontal gyrus, and the anterior insula when binge drinking college students were asked to perform alcohol-cued go/no-go task. Given that impairment in response inhibition has been suggested to contribute to substance use disorder (Zilverstand et al., 2018), several studies are investigating the effect of binge drinking on other frontal brain regions. For an extensive review of how alcohol consumption alters inhibitory control during adolescence see (Loeber and Duka, 2009; López-Caneda et al., 2012; Wetherill et al., 2013; Campanella et al., 2017; Blanco-Ramos et al., 2019). It is well-established that during adolescence risky behaviors such as alcohol and drug use increase (Eaton et al., 2012). Physiologically, these risk-taking behaviors have been attributed to the development and remodeling of reward-related neurocircuitry that continues to mature during adolescence (Galvan, 2010). Similarly, it has been determined that exposure to alcohol during the formative years enhances the motivation for alcohol through novelty-seeking with unintended consequences on motor function (White et al., 2000; Stansfield and Kirstein, 2007), due to alcohol-induced alterations in neurochemical markers in the prefrontal cortex. In attempts to establish the relationship between novelty seeking and motivation through human studies, Van Dyke and Fillmore (2015) used a cue reactivity paradigm that involves exposing drinkers to either images of beer or a series of non-drink images (i.e., food items), and then measuring motivation for operant response task. Reports from their experiment showed that alcohol-related cues increased operant response behavior in drinkers. The impact of adolescent binge drinking on reward and decision-making processes has also been reported in the literature. For example, by using the Iowa Gambling Task (IGT) as a decision-making parameter, a longitudinal study by Goudriaan et al. (2007) found an association between binge drinking and poor decision-making. Specifically, in their experiment, college students who were identified as stable high binge-drinkers made less advantageous choices on the gambling task than their low binge-drinking peers. In a recent study that used event-related potential (ERPs) and IGT to assess the impact of binge drinking on decision-making in female college students, Na et al. (2019) found a strong correlation between binge drinking and decision-making deficits. As shown previously, alterations to brain regions that mediate the decision-making process following binge alcohol consumption offer potential explanations to the deficits in decision-making observed in these two studies. Given the change in brain structure and neurocircuitry during adolescence, it is important to understand the impact of binge drinking on reward-driven behaviors. In a study that aimed to determine the potential impact of alcohol on reward processing in college binge drinkers, Cservenka et al. (2015) used a modified version of the Wheel of Fortune (WOF) coupled with functional magnetic resonance imaging. In their longitudinal study, Cservenka et al. (2015) found reduced cerebellar brain activity during reward processing in binge drinkers compared with the control group. The reduction in cerebellar brain activity in binge drinkers negatively correlated with the amount of alcohol consumed in the last 90 days (Cservenka et al., 2015). In another study that examines brain reactivity *via* a reward-guessing game (i.e., Win vs. Loss), Crane et al. (2017) found enhanced NAcc activation in their healthy binge drinking sample compared to the non-binge drinking group during reward processing relative to loss. As stated previously, the NAcc is critical for reward and sensation-seeking in adolescence (Morales et al., 2019), hence its enhanced activation in binge drinkers could be a risk factor for developing AUD later in life. Taken together, these results further support the hypothesis that binge alcohol consumption could cause structural and functional changes to frontal brain regions such as the superior frontal gyrus (SFG), inferior parietal lobule (IPL), and supramarginal gyrus (Squeglia et al., 2012), anterior cingulate cortex (Mashhoon et al., 2014; Cohen-Gilbert et al., 2017), cingulate gyrus (Norman et al., 2011), inferior frontal gyrus, and anterior insula (Suárez-Suárez et al., 2020), and other areas such as the cerebellum (Cservenka et al., 2015), and NAcc (Crane et al., 2017), potentially contributing to deficits in abilities such as decision making (Goudriaan et al., 2007), working memory (Campanella et al., 2013), verbal encoding (Schweinsburg et al., 2010), and inhibition response (Suárez-Suárez et al., 2020).

As stated previously, adolescent binge alcohol consumption leads to undesired neurobiological changes and exacerbates the risk of developing AUD during adulthood. Since many of the affected brain regions are involved in the modulation of reward and response to negative emotions, some studies are currently examining whether adolescent binge drinking reinforces further consumption and contributes to the development of AUD later in life. Experimentally, it has been determined that binge drinking at an early age enhances alcohol consumption in rodent models. For example, by using a four-day binge model, and analyzing dose and sex-related changes that occur when adolescent (PND 28) male and female Sprague Dawley rats are continuously administered different concentrations of ethanol, Maldonado-Devincci et al. (2010) observed that exposure to ethanol during adolescence pre-disposed the rats to consume more ethanol in adulthood. Currently, the use of operant tasks is being explored to enhance our understanding of the impact of adolescent binge drinking and alcohol use later in life. Alaux-Cantin et al. (2013) using Sprague-Dawley rats administered binge-like ethanol concentrations and demonstrated an association between early ethanol consumption and increased motivation to self-administer ethanol in young adults. Similar positive relationships between adolescent alcohol exposure in rats and increased alcohol consumption during adulthood have been reported in a recent experiment by Amodeo et al. (2017). In addition to increasing alcohol consumption later in life, it has also been shown that administering binge-like ethanol concentration (i.e., 3 g/kg) to adolescent rats by intraperitoneal injection can lead to cognitive deficits as demonstrated in the novel object recognition test (De Ferron et al., 2015). In a more recent study, Van Hees et al. (2022) demonstrated that adolescent mice that voluntarily binge drink alcohol *via* a modified drinking in the dark paradigm performed poorly on a novel object recognition test. In another study investigating the relationship between alcohol intermittent exposure and risk-seeking behaviors in rodents, Boutros et al. (2014) recorded a negative relationship between risky choices and the levels of dopamine, norepinephrine, and choline in alcohol-exposed rats. These experiments further support the premise that adolescent binge drinking reinforces further consumption and could contribute to the development of AUD later in life. In summary, through the human data and rodent adolescence models outlined here, there is enough evidence to demonstrate that binge alcohol consumption impairs cognitive functions such as visuospatial processing, working memory, learning, attention, inhibition response, and decision-making.

## 6. Discussion and future directions

Adolescence is a transitional period that is marked by biological changes that include brain maturation. Due to continued brain development during this stage, it is vulnerable to the effects of illicit drugs and alcohol which can lead to cognitive deficits later in life. The rate and amount of alcohol consumed by adolescents can be excessive (Adan et al., 2016; Hermens and Lagopoulos, 2018) and has been linked to cognitive dysfunction, behavioral conflict, unsafe sexual practices, vehicular accidents, and increased likelihood of developing AUD later in life (Hansson et al., 2010, Hong et al., 2013; Moure-Rodríguez et al., 2016; Hingson et al., 2017). Based on these concerns, many studies are currently exploring the impact of binge drinking on specific regions of the brain, especially areas that mediate higher cognitive functions (Crego et al., 2010; López-Caneda et al., 2012; Mashhoon et al., 2014). Through extensive research, it has been determined in both human and rodent models that binge alcohol consumption during adolescence disrupts white and gray matter development and normal function of cortical and hippocampal areas. Neuronal death and impaired adult neurogenesis appear to be consistently impacted across studies and likely involve the activation of non-neuronal cells and pro-inflammatory signaling. All of these factors likely contribute to alcohol-induced cognitive impairment that can persist into adulthood and can include the disruption of learning, attention, working memory, impulsivity, decision-making, and inhibition response. Even though significant steps have been taken to understand the impact of adolescent binge alcohol on neurocognitive function, future studies need to build on the knowledge gained from different fields of study. For example, using sequencing techniques to help understand the temporal changes that occur at the gene level during normal adolescent development and under the influence of chronic intermittent ethanol exposure is needed. These studies should be compared to GWAS data collected from patients and families with a history of AUD. Combined approaches to understand the fundamental changes in non-neuronal and neuronal cell interactions and responses to adolescent alcohol need to be rigorously addressed to further understand the cell-to-cell signaling mechanisms involved in the pro-inflammatory responses that occur acutely and during abstinence. Further work is needed to understand the drivers of alcohol-induced gray and white matter loss and how these changes impact the ongoing development of projecting neuronal circuitry, critical for higher cognitive function and reward-related behavior. Not surprisingly, comparative studies to further our understanding of sex differences need to be employed with the consideration of whether male optimized behavioral assays are also appropriately optimized for female rodent models of cognition. The development of new technologies, employing integrative methodologies, designing translationally relevant binge drinking paradigms in animal models that augment human studies, and examining cellular markers in a sex-specific manner would enhance our understanding of how binge drinking affects brain maturation during the formative years and leads to unintended neurocognitive complications.

## 7. Conclusion

The aim of this review is to discuss the maturational changes that occur in the adolescent brain and evaluate the effects of adolescent alcohol consumption on brain structure, function, and neurocognitive abilities in both human and animal models. As shown here, the adolescent brain undergoes important maturational changes necessary for effective brain development. However, at the same time, the brain also becomes susceptible to the neurotoxic effects of alcohol. During adolescence, negative impacts can emerge from peers, parents, and the environment, and contribute to increased binge alcohol consumption resulting in undesirable neurocognitive consequences. There is a need for more temporal, neuropathological studies on adolescent brain maturation across cell types with brain sub-region specificity that consider the impact of sex with and without binge alcohol exposure. There is no doubt that the knowledge gathered from both cross-sectional and translational studies will continue to provide a further understanding of the mechanisms that underlie the cognitive deficits that persist and/or emerge during abstinence. Continued investigation into this area of research will help create useful policies and clinical interventions to treat complications related to adolescent binge drinking across the lifespan.

## Author contributions

Both authors listed have made a substantial, direct, and intellectual contribution to the work, and approved it for publication.
